# *QuickStats:* Percentage* of Employed Adults Aged ≥18 Years Who Slept <7 Hours per 24-Hour Period,^†^ by Sex and Number of Work Hours per Week^§^ — United States, 2022

**DOI:** 10.15585/mmwr.mm7316a6

**Published:** 2024-04-25

**Figure:**
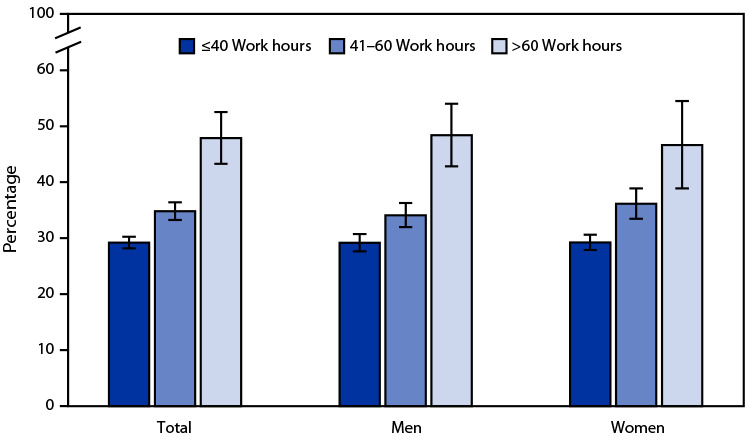
In 2022, the percentage of employed adults who slept <7 hours on average during a 24-hour period increased with the number of hours worked per week, including 29% among those who worked ≤40 hours, 35% among those who worked 41–60 hours, and 48% among those who worked >60 hours per week. The patterns were similar for men and women.

**Supplementary Table:** https://stacks.cdc.gov/view/cdc/153722

**Source:** National Center for Health Statistics, National Health Interview Survey, 2022. https://www.cdc.gov/nchs/nhis/index.htm

For more information on this topic, CDC recommends the following link: https://www.cdc.gov/sleep/about_sleep/sleep_hygiene.html

